# The tetrahedral structure and luminescence properties of Bi-metallic Pt_1_Ag_28_(SR)_18_(PPh_3_)_4_ nanocluster†

**DOI:** 10.1039/c6sc05104a

**Published:** 2017-01-05

**Authors:** Xi Kang, Meng Zhou, Shuxin Wang, Shan Jin, Guodong Sun, Manzhou Zhu, Rongchao Jin

**Affiliations:** a Department of Chemistry and Center for Atomic Engineering of Advanced Materials, Anhui University Hefei Anhui 230601 China zmz@ahu.edu.cn; b Department of Chemistry, Carnegie Mellon University Pittsburgh PA 15213 USA rongchao@andrew.cmu.edu

## Abstract

The atomic-structure characterization of alloy nanoclusters (NCs) remains challenging but is crucial in order to understand the synergism and develop new applications based upon the distinct properties of alloy NCs. Herein, we report the synthesis and X-ray crystal structure of the Pt_1_Ag_28_(S-Adm)_18_(PPh_3_)_4_ nanocluster with a tetrahedral shape. Pt_1_Ag_28_ was synthesized by reacting Pt_1_Ag_24_(SPhMe_2_)_18_ simultaneously with Adm-SH (1-adamantanethiol) and PPh_3_ ligands. A tetrahedral structure is found in the metal framework of Pt_1_Ag_28_ NC and an overall surface shell (Ag_16_S_18_P_4_), as well as discrete Ag_4_S_6_P_1_ motifs. The Pt_1_Ag_12_ kernel adopts a face-centered cubic (FCC) arrangement, which is observed for the first time in alloy nanoclusters in contrast to the commonly observed icosahedral structure of homogold and homosilver NCs. The Pt_1_Ag_28_ nanocluster exhibits largely enhanced photoluminescence (quantum yield QY = 4.9%, emission centered at ∼672 nm), whereas the starting material (Pt_1_Ag_24_ NC) is only weakly luminescent (QY = 0.1%). Insights into the nearly 50-fold enhancement of luminescence were obtained *via* the analysis of electronic dynamics. This study demonstrates the atomic-level tailoring of the alloy nanocluster properties by controlling the structure.

## Abstract 2

Tailoring the nanocluster at an atomic level leads to a tetrahedron-shaped FCC Pt_1_Ag_28_(S-Adm)_18_(PPh_3_)_4_ nanocluster and a large enhancement in photoluminescence.

## Graphical abstract

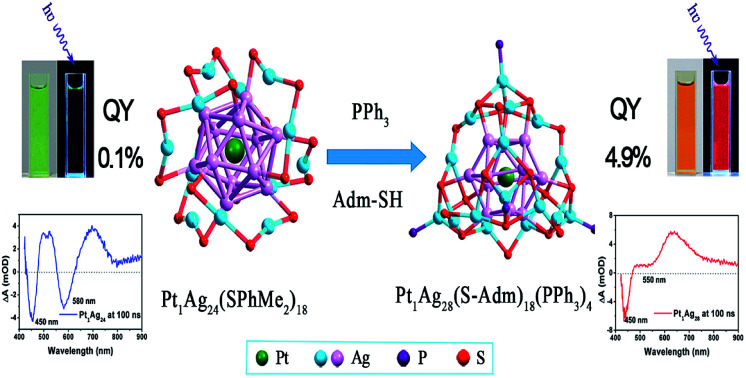

## 1 Introduction

Atomically precise metal nanoclusters (NCs) have attracted increasing interest as functional materials due to their distinct optical, catalytic, magnetic, and electrochemical properties.^1–7^ The well-defined structure of NCs with precise compositions permits atomic level structure–property correlations.^8–18^ To date, great progress has been achieved in the synthesis and characterization of NCs, including mono-metallic NCs (gold or silver) and alloy NCs.^19–26^ Among them, bi-metallic NCs may offer significantly enhanced properties compared to that of the single-component NCs due to the synergistic effects induced by the heteroatom(s).^20*b*,21*b*,22*b*,23,25*a*^ For example, drastically improved catalytic activity and enhanced luminescence (compared with the mono-metallic counterparts of NCs) have been achieved in M–Au (with a single dopant M = Pt or Pd)^27,28^ and Au–Ag bi-metallic NCs (with Au dopants),^21*b*,22*b*^ respectively. These results demonstrate the great potential of alloy NCs in catalytic, optical and biological applications.

Thus far, the atomic-level structural determination of bi-metallic NCs by X-ray crystallography has only been achieved in a few cases.^11,20*a*,21*b*,22–26^ The synthetic methods used to prepare bimetallic NCs can be roughly classified into two strategies: (1) the co-reduction of two metal precursors (*e.g.* complexes) in one-pot reactions^7,11,20,21*b*,22*b*,24^ and (2) doping mono-metallic NCs (which serve as templates) with heteroatom complexes.^25*a*,29–32^ For the synthesis of mono-metallic NCs, the thiol etching-induced transformation method is commonly used,^19,33^ which gives rise to NCs with novel structures and distinct properties. However, this etching strategy has not been applied to the synthesis of alloy NCs. The etching strategy is highly attractive for alloy NCs because the heteroatom(s) can be regarded as labelling atom(s), which provide mechanistic insights into the etching process (similar to the isotope tracing method used in molecular chemistry).

Herein, we report the attainment of Pt_1_Ag_28_ nanocluster co-protected by 1-adamantanethiolate (HS-Adm) and triphenyl phosphine (PPh_3_) ligands, formulated as Pt_1_Ag_28_(S-Adm)_18_(PPh_3_)_4_. The Pt_1_Ag_28_ nanoclusters are obtained by etching Pt_1_Ag_24_(SPhMe_2_)_18_ with both the HS-Adm and PPh_3_ ligands, and the crystal structure of Pt_1_Ag_28_ reveals that the Pt atom resides in the central position of the nanocluster. In addition, Pt_1_Ag_28_ shows unique structural features including: (1) the presence of a face-centered cubic (FCC) Pt_1_Ag_12_ kernel, which is observed for the first time in silver-based alloy NCs, as opposed to the common icosahedral structure, and (2) the discovery of new surface motifs, such as the Ag_4_(SR)_6_(PPh_3_)_1_ motif and its assembled cage-like structure that protects the FCC kernel. Furthermore, compared to the Pt_1_Ag_24_ precursor, the photoluminescence (PL) quantum yield (QY) of Pt_1_Ag_28_ is largely increased from 0.1% to 4.9% (*i.e.* about 50 times of enhancement) due to the suppressed relaxation of the excited state *via* phonon emission and other non-radiative pathways. Moreover, a significant enhancement in the thermal stability was also achieved in Pt_1_Ag_28_ compared to that of the Pt_1_Ag_24_ precursor nanocluster.

## 2 Experimental

### Materials

Hexachloroplatinic(iv) acid (H_2_PtCl_6_·6H_2_O, 99.99%, metals basis), silver nitrate (AgNO_3_, 99%, metals basis), 2,4-dimethylbenzenethiol (HSPhMe_2_, 99%), 1-adamantanethiol (C_10_H_16_S, 99%), triphenylphosphine (PPh_3_, 99%), tetraphenyl phosphonium bromide (PPh_4_Br, 98%) and sodium borohydride (NaBH_4_, 99.9%). Methylene chloride (CH_2_Cl_2_, HPLC grade, Aldrich), ethyl acetate (CH_3_COOC_2_H_5_, HPLC, Aldrich), methanol (CH_3_OH, HPLC, Aldrich) and *n*-hexane (Hex, HPLC grade, Aldrich). Pure water was purchased from Wahaha Co. Ltd. All reagents were used as received without further purification. All glassware were thoroughly cleaned with aqua regia (HCl : HNO_3_ = 3 : 1 vol%), rinsed with copious amounts of pure water and then dried in an oven prior to use.

### Synthesis of the [Pt_1_Ag_24_(SPhMe_2_)_18_](PPh_4_)_2_ nanocluster

For the nanocluster synthesis, AgNO_3_ (30 mg, 0.18 mmol) was dissolved in 5 mL of CH_3_OH and 15 mL of CH_3_COOC_2_H_5_. H_2_PtCl_6_·6H_2_O (4 mg, 0.0075 mmol) was dissolved in 5 mL of CH_3_OH and added to the reaction mixture. The resulting solution was vigorously stirred (about 1200 rpm) with a magnetic stirrer bar for 15 min. Then, 100 μL of HSPhMe_2_ was added. After another 15 min, 1 mL of NaBH_4_ aqueous solution (20 mg mL^−1^) was added quickly to the reaction mixture under vigorous stirring. The reaction was allowed to proceed for 24 hours under a N_2_ atmosphere. After the reaction was complete, the mixture in the organic phase was rotavaporated under vacuum, and then 20 mL of CH_3_OH was used to extract the product, which also contained the redundant HSPhMe_2_ and by-products. 5 mL of a CH_3_OH solution containing excess PPh_4_Br was added into the abovementioned CH_3_OH solution. Subsequently, the resulting solution was centrifuged to obtain the solid. Approximately 15 mL of methanol was added to wash the synthesized nanocluster. The precipitate was then dissolved in CH_2_Cl_2_ giving rise to [Pt_1_Ag_24_(SPhMe_2_)_18_](PPh_4_)_2_ nanoclusters (34 mg, 0.006 mmol, yield: 80.5% on a Ag mole basis).

### Synthesis of Pt_1_Ag_28_(S-Adm)_18_(PPh_3_)_4_ nanocluster

For the nanocluster synthesis, 10 mg of (PPh_4_)_2_[Pt_1_Ag_24_(SPhMe_2_)_18_] was dissolved in 10 mL of CH_2_Cl_2_. Then, 5 mg of PPh_3_ and 10 mg of AdmSH were added to the solution simultaneously. The reaction was allowed to proceed for 30 min at room temperature. The colour of solvent transformed from bright green to orange. The organic layer was separated from the precipitate and evaporated to dryness. The Pt_1_Ag_28_(S-Adm)_18_(PPh_3_)_4_ nanocluster was obtained afterwards. The dried nanocluster was washed with methanol at least 3 times and collected by centrifugation (7 mg, 0.001 mmol, yield: 63.2% on a Ag mole basis).

### Characterization

All UV-vis absorption spectra of the nanoclusters dissolved in CH_2_Cl_2_ were recorded using an Agilent 8453 diode array spectrometer, whose background correction was made using a CH_2_Cl_2_ blank. Solid samples were dissolved in CH_2_Cl_2_ to make a dilute solution with its subsequent transformation to a 1 cm path length quartz cuvette, which was followed by the spectral measurements. Thermogravimetric analysis (TGA) was carried out on a thermogravimetric analyzer (DTG-60H, Shimadzu Instruments, Inc.) with 5 mg of nanocluster in a SiO_2_ pan at a heating rate of 10 K min^−1^ from room temperature (about 298 K) to 1073 K. X-ray photoelectron spectroscopy (XPS) measurements were performed on a Thermo ESCALAB 250, configured with a monochromated Al Kα (1486.8 eV) 150 W X-ray source, 0.5 mm circular spot size, a flood gun to counter charging effects, and a analysis chamber base pressure lower than 1 × 10^−9^ mbar; data were collected at FAT = 20 eV. Photoluminescence spectra were measured on a FL-4500 spectrofluorometer with the same optical density (OD) ∼0.05. In these experiments, the nanocluster solutions were prepared in CH_2_Cl_2_ at a concentration of less than 1 mg mL^−1^. Absolute quantum yields (QY) were measured using dilute solutions of the clusters (0.05 OD absorption at 480 nm) on a HORIBA FluoroMax-4P. Inductively coupled plasma-atomic emission spectrometry (ICP-AES) measurements were performed on an Atomscan Advantage instrument made by Thormo Jarrell Ash Corporation (USA). The nanoclusters were digested with concentrated nitric acid and the concentration of the nanoclusters was set to ∼0.5 mg L^−1^.

### Single-crystal growth and analysis

Single crystals of the Pt_1_Ag_28_(S-Adm)_18_(PPh_3_)_4_ nanocluster were grown at 4 °C for 2–3 days in CH_2_Cl_2_/hexane. Red crystals were collected and the structures of Pt_1_Ag_28_(S-Adm)_18_(PPh_3_)_4_ were determined. The data collection for single crystal X-ray diffraction was carried out on a Bruker Smart APEX II CCD diffractometer under a liquid nitrogen flow at 150 K using graphite-monochromatized Cu Kα radiation (*λ* = 1.54178 Å). Data reductions and absorption corrections were performed using the SAINT and SADABS programs, respectively. The structure was solved using direct methods and refined with full-matrix least squares on *F*^2^ using the SHELXTL software package. All non-hydrogen atoms were refined anisotropically, and all the hydrogen atoms were set in geometrically calculated positions and refined isotropically using the riding model.

### Femto-nanosecond transient absorption spectra

Details of the femtosecond experiments have been described elsewhere.^37^ Nanosecond transient absorption spectra were measured based on the same ultrafast pump pulses along with an electronically delayed supercontinuum light source with a sub-nanosecond pulse duration (EOS, Ultrafast Systems).

### Time-correlated single-photon counting

Fluorescence lifetimes were measured using a time-correlated single photon counting (TCSPC) technique (Fluorolog-3 HORIBA Jobin Yvon); a pulsed LED source (376 nm, 1.1 ns) was used as the excitation source. The instrument response function (IRF) of detection was about 1.5 ns.

## 3 Results and discussion

### Characterization of the reaction

**Fig. 1:**
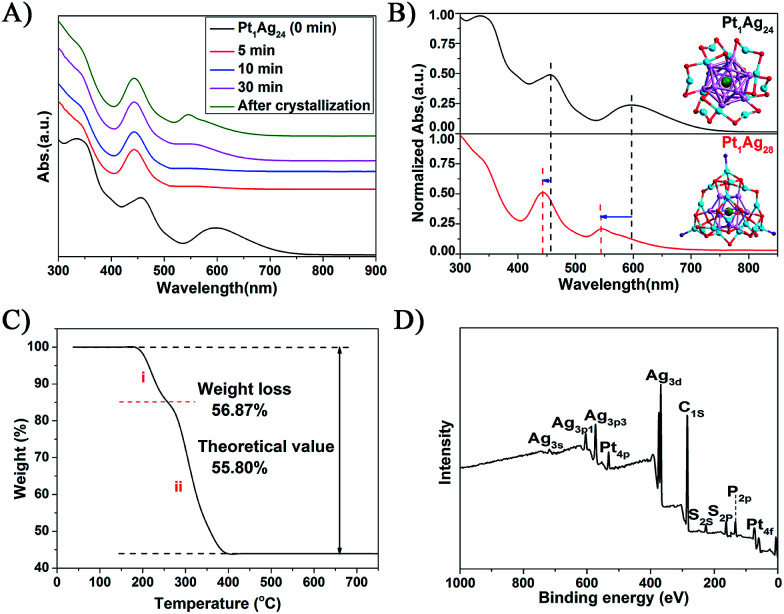
(A) The evolution of the etching of Pt_1_Ag_24_(SR)_18_ with co-present AdmSH and PPh_3_ ligands. (B) The UV-vis spectral comparison of the Pt_1_Ag_24_ and Pt_1_Ag_28_ NCs. Insets: the X-ray crystal structures of Pt_1_Ag_24_ and Pt_1_Ag_28_. Color codes: green spheres = Pt; cerulean sphere = Ag on the shell; violet sphere = Ag in the kernel; red sphere = S; purple sphere = P; carbon tails are omitted for clarity. (C) TGA of Pt_1_Ag_28_. (D) XPS spectrum of Pt_1_Ag_28_.

The reaction was monitored *via* UV-vis spectroscopy (Fig. 1A), in which the spectra show the gradual conversion of Pt_1_Ag_24_(SR)_18_ to Pt_1_Ag_28_(S-Adm)_18_(PPh_3_)_4_ when reacting with Adm-SH and PPh_3_ together as reflected in the spectral changes. For a close comparison, the spectra of Pt_1_Ag_24_ and the etching product Pt_1_Ag_28_ are shown in Fig. 1B, in which one can see that the 465 and 600 nm absorption bands of Pt_1_Ag_24_ are blue-shifted to 445 and 545 nm, respectively, after the conversion. In addition, we tested Adm-SH as the sole etching reagent in this reaction. The product was a mixture of larger nanoclusters as opposed to pure Pt_1_Ag_28_ (Fig. S2†).

Thermogravimetric analysis (TGA) of the product shows a total weight loss of 56.8 wt% (Fig. 1C), which is consistent with the theoretical loss (55.8 wt%) according to the formula determined *via* X-ray crystallography (*vide infra*); it should be noted that the loss of PPh_3_ ligands accounts for 15.2 wt% and S-Adm ligands for 41.6 wt% (Fig. 1C), which is also consistent with the theoretical losses (14.4 and 41.4 wt%, respectively). The atomic ratio of Pt to Ag was analyzed *via* inductively coupled plasma (ICP) atomic emission spectroscopy to be Pt/Ag = 3.9/96.1 and also by X-ray photoelectron spectroscopy (XPS) to be Pt/Ag = 3.5/96.5, which are consistent with the expected ratio of Pt/Ag = 3.5/96.5 (see Fig. 1D, S3 and Table S1† for the data and details). Electrospray ionization mass spectrometry (ESI-MS) confirmed the purity of Pt_1_Ag_28_ (Fig. S4†), in the results of which only one peak was found (*m*/*z* = 3637.67 Da, with *z* = 2+ and perfectly matched the experimental and simulated isotope patterns of [Pt_1_Ag_28_(SR)_18_(PPh_3_)_4_]^2+^).

### Atomic structure

**Fig. 2:**
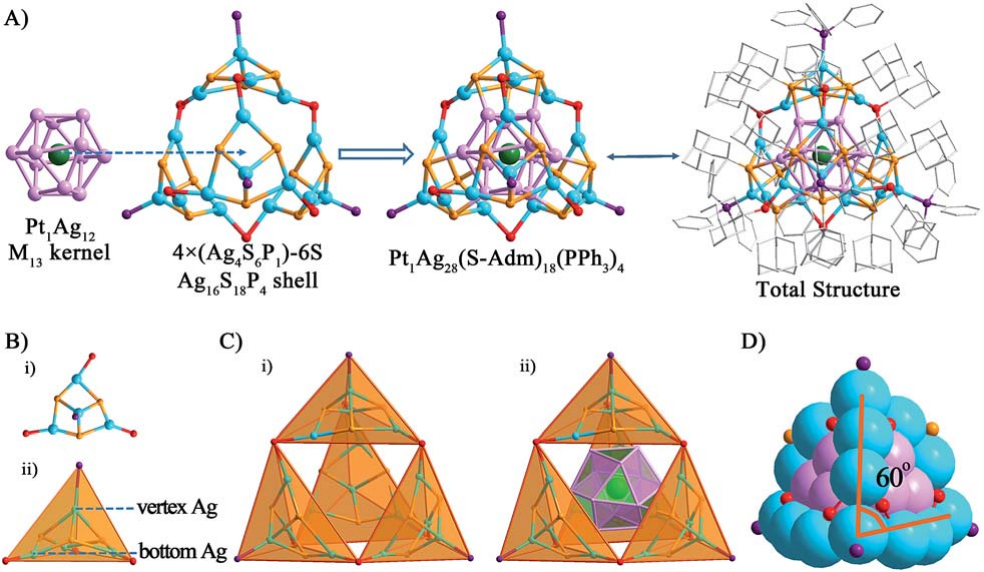
Ball-and-stick views of (A) the Pt_1_Ag_12_ kernel, outer-motif and the overall structure; (B) the Ag_4_S_6_P_1_ motif in the tetrahedral shape and (C) the total structure in the tetrahedral shape. (D) A space-filling view of the nanocluster. Color codes: green spheres, Pt; cerulean sphere, Ag on the shell; violet sphere, Ag in the kernel; orange sphere, S bonding the kernel; red sphere, S linking the motifs; purple sphere, P. For clarity, the hydrogen atoms are not shown.

The structure of Pt_1_Ag_28_ can be divided into two parts, the kernel and the surface shell. By comparing the crystal structures of the Pt_1_Ag_24_ and Pt_1_Ag_28_ nanoclusters, we identified that the six Ag_2_S_3_ (–R groups omitted) staple motifs surrounding the Pt_1_Ag_12_ kernel in the Pt_1_Ag_24_ nanocluster change to the four Ag_4_S_6_P_1_ motifs sharing six S atoms, forming an overall Ag_16_S_18_P_4_ shell in a tetrahedral shape with the 4 motifs at the 4 vertices of tetrahedron (Fig. 2). As for the kernel structure, the icosahedral Pt_1_Ag_12_ kernel of Pt_1_Ag_24_ was converted into an FCC kernel (*vide infra*). The single Pt atom is surrounded by an Ag_12_ shell to form the Pt_1_Ag_12_ kernel. The Pt_1_Ag_12_ kernel was further encircled by an integrated Ag_16_S_18_P_4_ cage-like exterior shell. Thus, the entire structure shows a tri-stratified arrangement—Pt(center)@Ag_12_(shell)@Ag_16_S_18_P_4_ (exterior). The bond lengths and bond angles are given in the ESI (Table S2†).

For a better view, the overall Ag_16_S_18_P_4_ shell was dissected into four equivalent Ag_4_S_6_P_1_ motifs sharing six S atoms. The six S atoms in each Ag_4_S_6_P_1_ motif are divided into two forms (Fig. 2A and B): (1) the three S atoms (in red, vertically linking to the Ag atoms) bond to the Ag atoms in the kernel–shell (3 × 4 = 12Ag atoms in the M_13_ kernel), which can be regarded as the bridges between the kernel and motif outside. The total 12S atoms stabilize the M_13_ kernel in the overall structure; (2) the other three S atoms (in orange, connecting the bottom Ag atoms) act as linkers to connect two nearby Ag_4_S_6_P_1_ motifs to form the integrated motif shell—Ag_16_S_18_P_4_. Interestingly, the P atom and three bottom S atoms in each Ag_4_S_6_P_1_ motif constitute a tetrahedral structure. The overall Ag_16_S_18_P_4_ shell consists of four Ag_4_S_6_P_1_ tetrahedral motifs (Fig. 2C), and the integrated configuration is also approximately a tetrahedron. The overall metal framework of Pt_1_Ag_28_ (Fig. 2D) also adopts a tetrahedral shape, which is constructed by six 4-atom-long edges (Ag atoms from the shell) and four faces of Ag_3_ (Ag atoms from the kernel). In addition, the angle between the edges is approximately 60° (Fig. 2D), which is consistent with the standard tetrahedral structure.

**Fig. 3:**
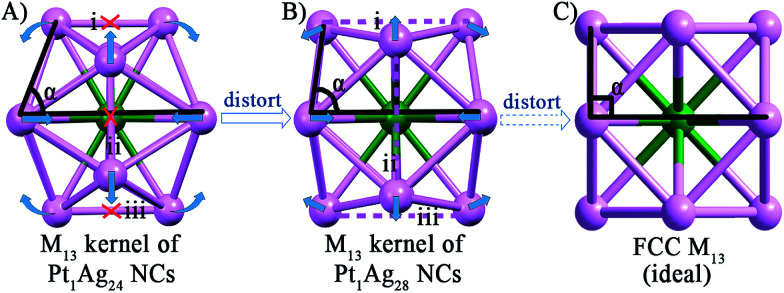
The Pt_1_Ag_12_ kernels of the (A) Pt_1_Ag_24_ and (B) Pt_1_Ag_28_ NCs. (C) The standard FCC M_13_ kernel. Color codes: green sphere, Pt; violet sphere, Ag.

As to the structure of the Pt_1_Ag_12_ kernel, the icosahedral kernel of Pt_1_Ag_24_ was transformed to the FCC arrangement in Pt_1_Ag_28_ after the etching process. To the best of our knowledge, all the previously reported M_13_ (M = Au/Ag/Pt) kernels are icosahedral, and thus the FCC arrangement in the M_13_ kernel was observed for the first time in an alloy NC. Based on the well-defined structure of Pt_1_Ag_24_ and Pt_1_Ag_28_, we propose a plausible mechanism for the transformation process. As shown in Fig. 3, the Pt_1_Ag_12_ kernel of Pt_1_Ag_24_ is in an icosahedral arrangement by the triangular shape of each face. When etched with Adm-SH and PPh_3_ ligands, the relative positions of the Ag atoms on the kernel's surface shift, and consequently the bonds of the Pt_1_Ag_12_ kernel in Pt_1_Ag_24_ (*i.e.* bonds i–iii in Fig. 3A, 2.960–2.991 Å) stretch to ∼3.610 Å, indicating that the Ag–Ag bonds were broken. Simultaneously, the angle *α* enlarges from 73.6° to 84.7°. Thus, some of the triangular faces in the kernel of Pt_1_Ag_24_ are re-arranged into a quadrilateral in Pt_1_Ag_28_ and the kernel is thus converted from an icosahedral to FCC arrangement. Furthermore, the slight distortion of the Pt_1_Ag_12_ kernel in Pt_1_Ag_28_ compared to the typical FCC M_13_ kernel (Fig. 3C) was caused by the interaction between the kernel and the outside motifs.

As mentioned earlier, the heteroatom(s) can be used as labeling atom(s) to shed light on the mechanism of the structural transformation. In this study, the central position of the Pt atom (as the only heteroatom) was retained during the etching process. This phenomenon indicates that the M_13_ kernel of Pt_1_Ag_24_ does not fall apart in the etching process and instead it just becomes distorted in response to the transformation of the exterior motifs. On the other hand, the stability of the central Pt atom was also established in the Pt_1_Ag_24_ and Pt_1_Au_24_ cases using density functional theory (DFT) calculations and experimental studies.^20*b*,28^

Recently, an Ag_29_ NC co-protected with BDT (1,3-benzenedithiol) and PPh_3_ ligands as well as a single Au doped Au_1_Ag_28_ NC have been reported by Bakr and co-workers.^22^ In the present study, we discovered that the framework of Pt_1_Ag_28_ was largely different from Ag_29_ and Au_1_Ag_28_, albeit all of them have the same metal atom number (*i.e.* 29). The distinct differences in Pt_1_Ag_28_ compared to the other two examples are manifested in the following (see Fig. S5–8†): (1) the kernels of Ag_29_ and its Au-doped alloy are an icosahedral M_13_, whereas Pt_1_Ag_28_ possesses an FCC Pt_1_Ag_12_ kernel; (2) the motifs on the nanocluster surface are also entirely different; the Ag_29_ (or Au_1_Ag_28_) NC possesses four Ag_1_S_3_P_1_ and four Ag_3_S_3_ motifs (carbon tails omitted), whereas the Pt_1_Ag_28_ is comprised of four new Ag_4_S_6_P_1_ motifs. By sharing six thiolates, the four Ag_4_S_6_P_1_ motifs form a cage-like Ag_16_S_18_P_4_ structure; (3) in the Pt_1_Ag_28_ nanocluster, all the metal atoms are located within the tetrahedron constructed *via* the four P atoms, while in Ag_29_ and Au_1_Ag_28_, 12Ag atoms out of the 29 metal atoms overflow the corresponding tetrahedron (*i.e.*, only 17 atoms are completely contained in the tetrahedron). In addition, it should be noted that tetrahedron-shaped Au NCs have been studied previously^34^ and our study fills in the blank in tetrahedral Ag NCs; (4) several smaller tetrahedral units were found in Pt_1_Ag_28_, such as the Ag_4_S_6_P_1_ motifs, the assembled motif structure and the overall metal nanocluster structure; however, such tetrahedrons are not observed in the Ag_29_ and Au_1_Ag_28_ NCs; (5) a charge state of −3 was reported for Ag_29_ and Au_1_Ag_28_ with an electron count of 8e (that is, 29 − 24 + 3 = 8e). In Pt_1_Ag_28_, ESI-MS (Fig. S4†) identified that the cluster bears 2+ charges (not 3− in Ag_29_ and Au_1_Ag_28_), but the X-ray crystallographic analysis did not find any counter ion (presumably Cl^−^ disordered in the crystal). Taking the results together, the nominal electron count of Pt_1_Ag_28_ is 8e (that is, 28 − 18 − 2 = 8e).

#### The optical energy gaps and photoluminescence properties

**Fig. 4:**
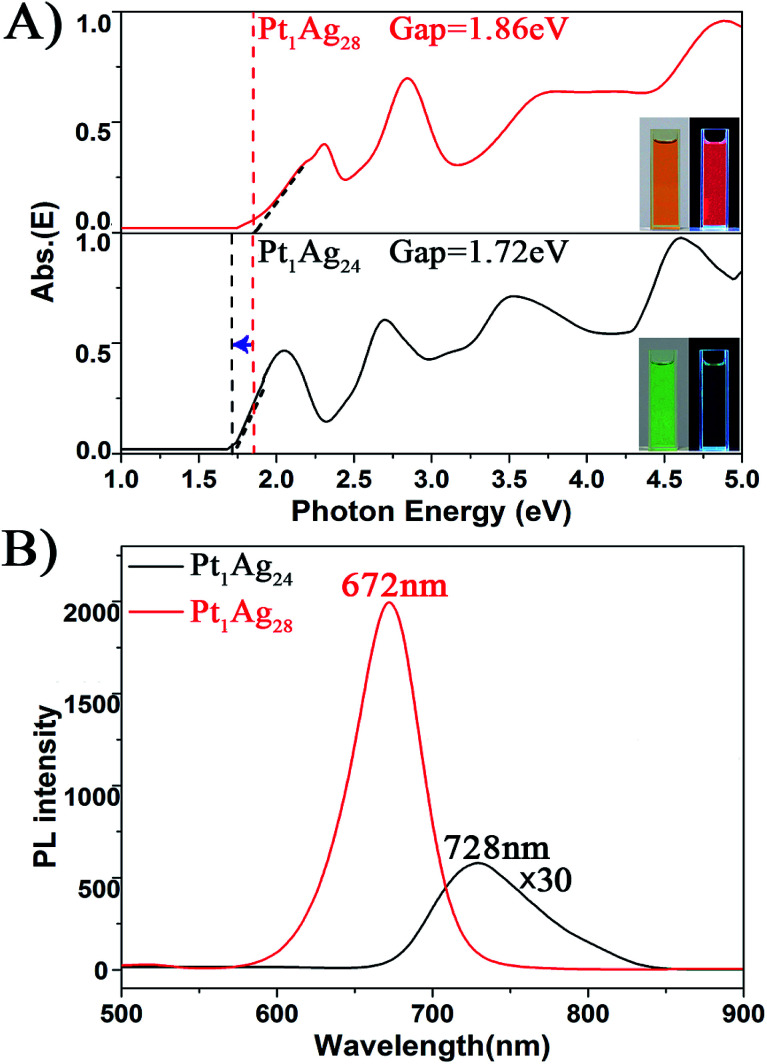
The spectra on the (A) energy scale and (B) PL of the Pt_1_Ag_24_ and Pt_1_Ag_28_ NCs. Insets of (A): digital photographs of each nanocluster in CH_2_Cl_2_ solution under visible and UV light.

To further compare the properties of the Pt_1_Ag_28_ with Pt_1_Ag_24_, the absorption spectra and PL of both samples were analyzed. The energy-scale absorption spectra of Pt_1_Ag_24_ and Pt_1_Ag_28_ are shown in Fig. 4A, with the optical energy gap of Pt_1_Ag_24_ being 1.72 eV and Pt_1_Ag_28_ being 1.86 eV. To the naked eye, the solution color of Pt_1_Ag_24_ is green, whereas the solution color of Pt_1_Ag_28_ is orange (insets of Fig. 4A). The trend of the optical gap energies was a surprise since typically one would expect the larger size of Pt_1_Ag_28_ to have a smaller gap than that of Pt_1_Ag_24_.

With respect to the PL properties (Fig. 4B), the QY of Pt_1_Ag_24_ is very low (0.1%),^20*b*^ but interestingly the QY is largely increased to 4.9% in Pt_1_Ag_28_ (by about a 50-fold enhancement). The PL of Pt_1_Ag_28_ is strong enough to be perceived by the naked eye. In addition, the PL peak of Pt_1_Ag_24_ was centered at 728 nm but it blue-shifts to 672 nm in Pt_1_Ag_28_ (a shift of *ca.* 56 nm). The PL excitation spectrum of Pt_1_Ag_28_ was also measured, which is almost identical to its absorption spectrum (Fig. S9†), indicating typical quantum confinement behavior and electron relaxation to the LUMO level before fluorescing.

#### The excited state behavior of Pt_1_Ag_24_ and Pt_1_Ag_28_

**Fig. 5:**
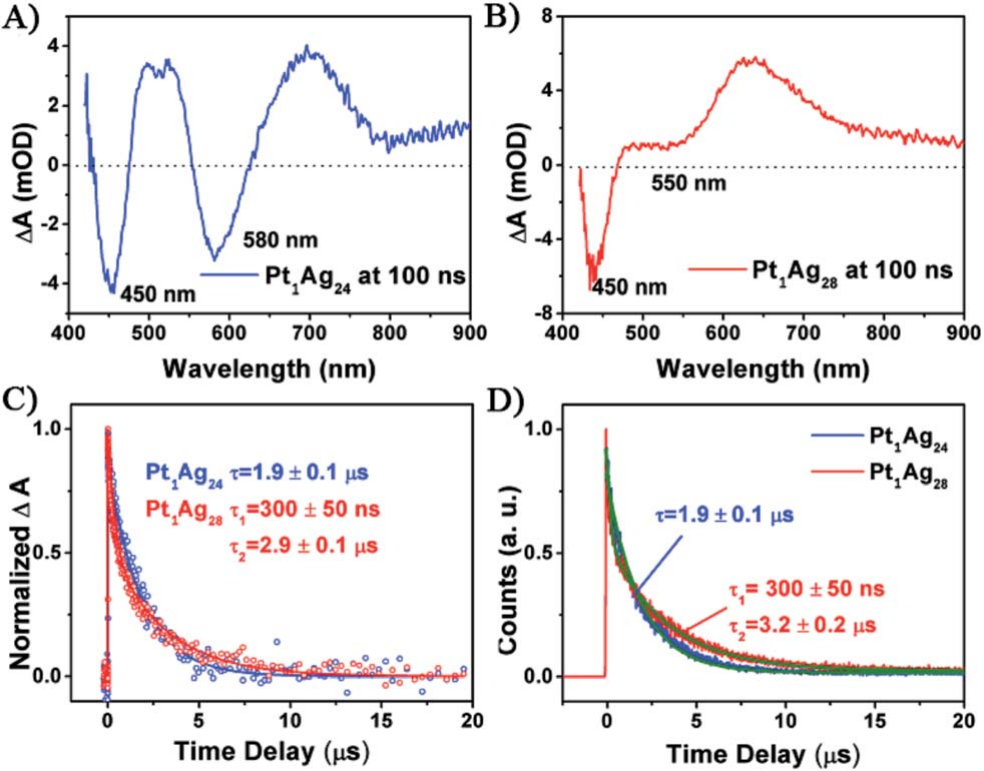
The transient absorption spectra at a time delay = 100 ns for (A) Pt_1_Ag_24_ and (B) Pt_1_Ag_28_ with excitation at 360 nm. (C) Normalized kinetic traces obtained from the transient absorption of both nanoclusters. (D) Normalized kinetic traces of fluorescence decay of both clusters at the emission maxima measured using TCSPC. All the solid lines are fits of the data.

Femto-nanosecond transient absorption spectroscopy (fs–ns TA) and time-correlated single-photon counting (TCSPC) were performed on both the Pt_1_Ag_24_ and Pt_1_Ag_28_ nanoclusters in order to probe their excited state properties. Upon excitation at 360 nm, Pt_1_Ag_24_ exhibits net ground state bleaching (GSB) at 450 nm and 580 nm, which corresponds to the UV-vis absorption and excited state absorption (ESA) around 500 nm and 700 nm (Fig. 5A). For Pt_1_Ag_28_ (Fig. 5B), the TA spectra exhibit an ESA centered at 650 nm, a net GSB around 450 nm and a dip around 550 nm, which also agrees with the steady state absorption. The kinetic traces of the GSB around 450 nm for both nanoclusters were fitted and compared (Fig. 5C).

For Pt_1_Ag_24_, the kinetic traces can be well fitted by a single exponential decay with a time constant of 1.9 μs, whereas for Pt_1_Ag_28_, two exponential decays (300 ns, 2.9 μs) were required to obtain the best fitting quality. Further femtosecond transient absorption measurements were performed, and the ultrafast relaxation dynamics for both clusters indeed exhibited similar behavior, which contain an ultrafast and a long lived decay (Fig. S10†). The fluorescence decays measured using time-correlated single-photon counting (TCSPC) exhibit lifetimes similar to that obtained from ns-TA measurements (*cf.*Fig. 5C and D), which suggests that the decay components in Pt_1_Ag_24_ and Pt_1_Ag_28_ clusters are all radiative.

The long-lived decay in silver nanoclusters has been ascribed to ligand to metal charge transfer (LMCT) but the origin of which is not fully understood.^21*b*,22*b*,35–37^ As the crystal structures of Pt_1_Ag_24_ and Pt_1_Ag_28_ are different, it would be helpful to compare the excited state behavior between the homosilver Ag_25_ and Ag_29_ nanoclusters with their Pt doped counterparts, which have similar structures.^21*a*,22*a*,38^ Table S3† lists the excited state lifetimes of Ag_25_ and Ag_29_ from the literature together with those of Pt_1_Ag_24_ and Pt_1_Ag_28_ obtained in this study. Both Ag_25_ and Ag_29_ have relatively low fluorescence (QYs < 1%).^21*a*,22*a*^ From Ag_25_ to Pt_1_Ag_24_, the lifetime slightly increases from 1.1 μs (ref. 21b) to 1.9 μs, whereas from Ag_29_ to Pt_1_Ag_28_ the lifetime increases from 300 ns (ref. 22b) to dual lifetimes (300 ns and 3.3 μs). The more drastic change in lifetime from Ag_29_ to Pt_1_Ag_28_ suggests that the electronic structure is more strongly modified in the case of Pt_1_Ag_28_, which may enhance the LMCT and lead to a higher fluorescence quantum yield. Moreover, prominent coherent oscillations were observed in the femtosecond kinetic traces of Pt_1_Ag_24_, whereas no such phenomenon was observed for Pt_1_Ag_28_ (Fig. S11†). The stronger phonon emission observed in Pt_1_Ag_24_ suggests that more excited-state energy is dissipated into the environment through heat, which also explains its weaker luminescence than that of Pt_1_Ag_28_.

### Thermal stability

**Fig. 6:**
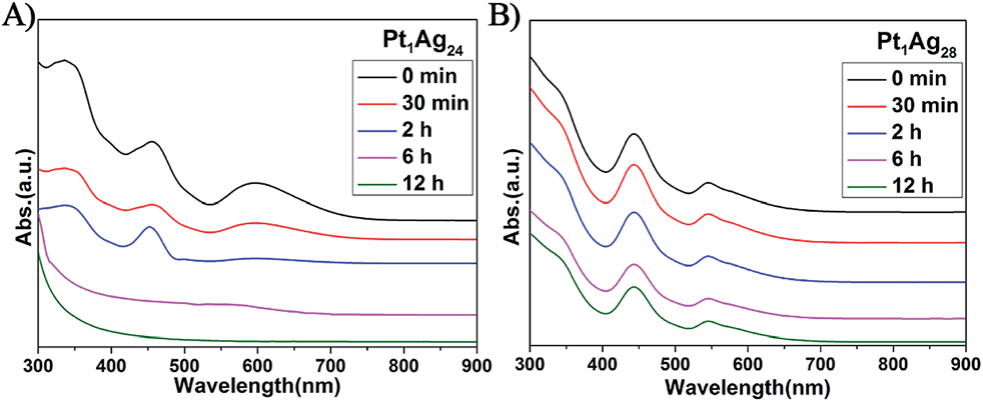
UV/Vis spectra confirming the thermal stability at 50 °C of the (A) Pt_1_Ag_24_ and (B) Pt_1_Ag_28_ NCs over time.

In addition to the PL properties, we further investigated the stability of Pt_1_Ag_24_ and its etching product, Pt_1_Ag_28_ (Fig. 6). The stability of these NCs was tested at 50 °C in air (NCs dissolved in CHCl_3_). As to Pt_1_Ag_28_, the UV-vis spectra were essentially unchanged over time (12 hours tested), which indicates its high stability, whereas the UV-vis spectra of Pt_1_Ag_24_ significantly decrease in intensity after two hours and completely disappear in approximately six hours. The higher thermal stability of Pt_1_Ag_28_ than that of Pt_1_Ag_24_ was ascribed to the more robust tetrahedral structure than the icosahedral one of Pt_1_Ag_24_.

The stability of Pt_1_Ag_24_ and Pt_1_Ag_28_ was also characterized *via* TGA measurements. As depicted in Fig. S12b and d,† the maximum weight loss temperature of Pt_1_Ag_24_ was 240 °C (*i.e.* the derivative curve), which was much lower than that of Pt_1_Ag_28_ (310 °C). It should be noted that the 220 °C peak in Pt_1_Ag_28_ corresponds to the loss of PPh_3_ ligands, which are easier to lose compared to the thiolate ligands. Bakr and co-workers reported that the lack of PPh_3_ ligands did not alter the overall configuration of Ag_29_ and Au_1_Au_28_.^22^ Thus, we suspect that the configuration of Pt_1_Ag_28_ was maintained at this stage. These results indicate higher stability of Pt_1_Ag_28_ compared to that of Pt_1_Ag_24_.

## 4 Conclusion

In summary, we devised an etching method for the conversion of Pt_1_Ag_24_(SPhMe_2_)_18_ to Pt_1_Ag_28_(S-Adm)_18_(PPh_3_)_4_ in the presence of Adm-SH and PPh_3_. The central Pt atom is retained in the conversion process; however, the Pt_1_Ag_12_ kernel was converted from an icosahedron to FCC arrangement, which is observed for the first time in the M_13_ kernel of alloy NCs. Multiple tetrahedral motifs were identified in the alloy NC, such as the Ag_4_S_6_P_1_ surface motif, the integrated motif shell Ag_16_S_18_P_4_, and the overall metal framework. The PL QY is significantly increased from only 0.1% for Pt_1_Ag_24_ to 4.9% for Pt_1_Ag_28_ (about a 50-fold enhancement). The ultrafast dynamics results reveal that the enhanced luminescence of Pt_1_Ag_28_ was due to the suppressed phonon emission and other non-radiative pathways in the tetrahedral structure. In addition, the thermal stability of Pt_1_Ag_28_ was drastically enhanced compared to that of its precursor, Pt_1_Ag_24_. It is hoped that this study will help stimulate the future discovery of new alloy NCs with tailored functionalities for wide applications in sensing and energy fields.

## Supplementary Material

SC-008-C6SC05104A-s001

SC-008-C6SC05104A-s002
