# Supplementary material for: The tetrahedral structure and luminescence properties of Bi-metallic Pt1Ag28(SR)18(PPh3)4 nanocluster
Source: Chem Sci. 2017 Jan 5;8(4):2581–7. doi: 10.1039/c6sc05104a (PMC5431686; doi:10.1039/c6sc05104a)
Supplement: SC-008-C6SC05104A-s001 [file SC-008-C6SC05104A-s001.pdf]

Electronic Supplementary Material (ESI) for Chemical Science.
This journal is © The Royal Society of Chemistry 2017

Supporting Information:

Tetrahedral Structure and Luminescence Properties of Bi-Metallic $Pt_1Ag_{28}(SR)_{18}(PPh_3)_4$ Nanocluster

Xi Kang,[a] Meng Zhou,[b] Shuxin Wang,[a] Shan Jin,[a] Guodong Sun,[a] Manzhou Zhu[a,*] & Rongchao Jin[b,*]

[a]Department of Chemistry and Center for Atomic Engineering of Advanced Materials, Anhui University, Hefei, Anhui 230601, China.
[b]Department of Chemistry, Carnegie Mellon University, Pittsburgh, Pennsylvania 15213, United States.

Structural analyses of Pt_1Ag_{28}, Au_1Ag_{28} and Ag_{29}

Structurally, the previous studies suggested that the structure and composition of the metal core significantly affect the M-S bond lengths. Thus the bond lengths and angles in Pt_1Ag_{28} were compared with the related ones in Ag_{29} and Au_1Ag_{28} (Table S2). Compared the bond lengths in Pt_1Ag_{28} with Ag_{29} and Au_1Ag_{28}, the range is larger (Au_1Ag_{28} is similar with Ag_{29} owing to the almost identified framework) although the average bond length is very similar. Regarding to the angles of P-Ag-S on the same motifs, the average angles of Ag_{29} and Pt_1Ag_{28} is 106.91^o and 105.94^o, respectively. However the average angles in Pt_1Ag_{28} is 126.80, which varies substantially with Ag_{29} (Au_1Ag_{28}).

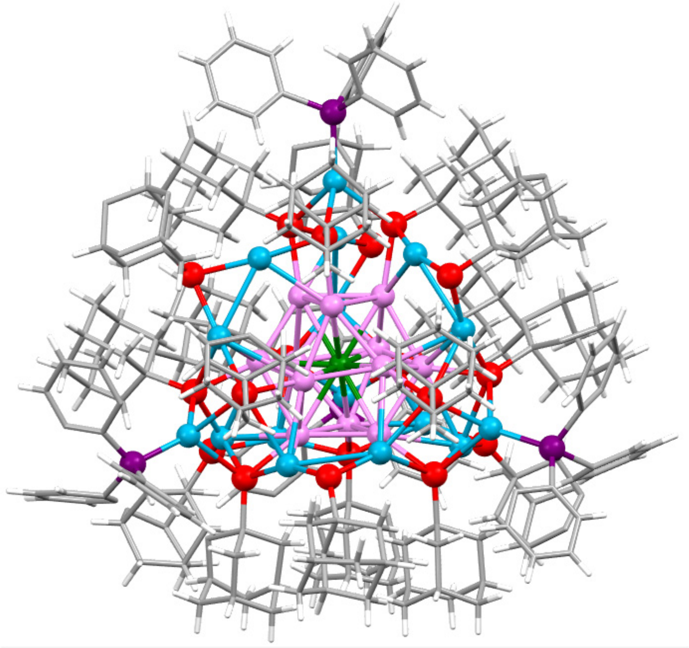

Fig. S1. The total structure of $Pt_1Ag_{28}(S\text{-}Adm)_{18}(PPh_3)_4$. Color legend: green sphere, Pt; cerulean sphere, Ag on the shell; violet sphere, Ag in the kernel; red sphere, S; purple sphere, P. For clarity, C and H are shown in wireframe.

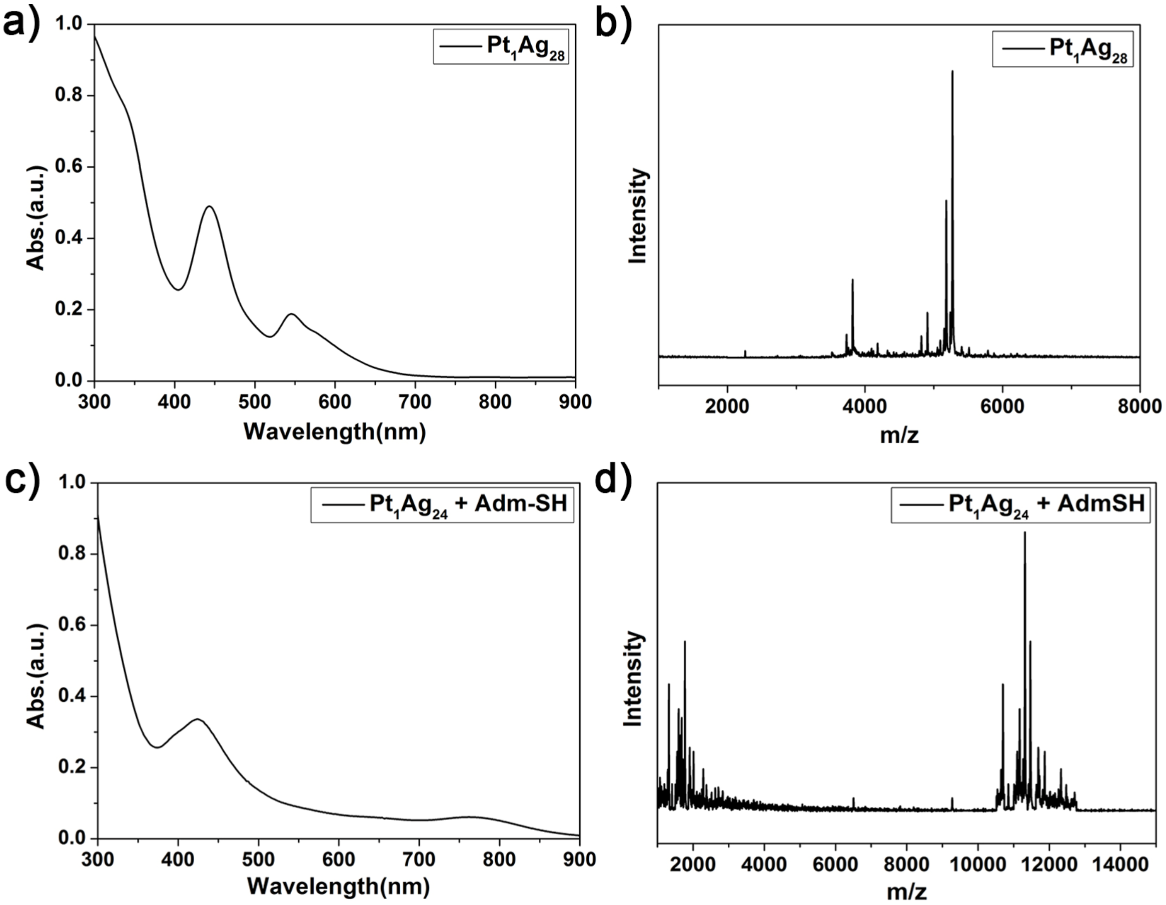

Fig. S2. a) UV-Vis spectrum of Pt_1Ag_{28}; b) MALDI-MS spectrum of Pt_1Ag_{28}; c) UV-Vis spectrum of Pt_1Ag_{24} etching by Adm-SH only ; d) MALDI-MS spectrum of Pt_1Ag_{24} etching by Adm-SH only.

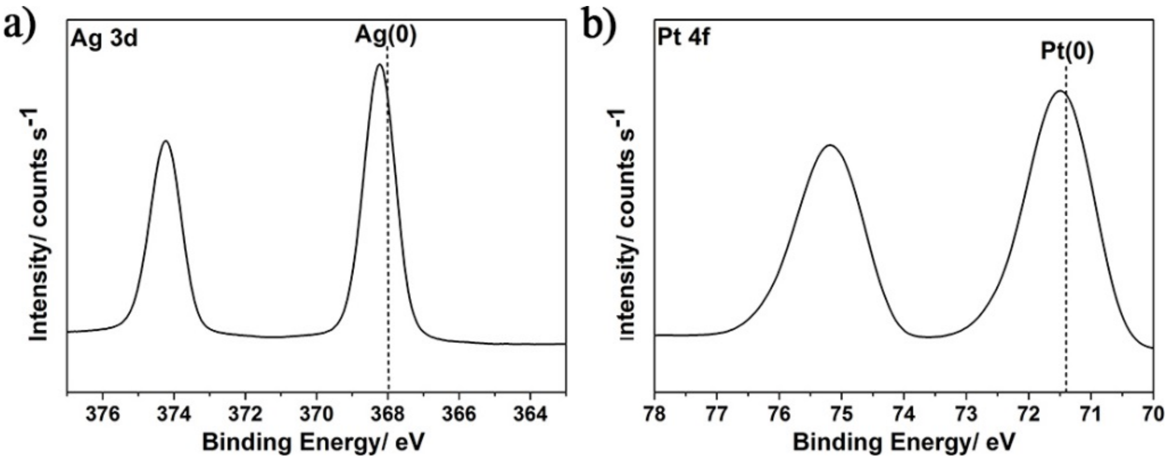

Fig. S3. X-ray photoelectron spectroscopy (XPS) of a) Ag 3d, b) Pt 4f in $Pt_1Ag_{28}(S\text{-}Adm)_{18}(PPh_3)_4$ nanocluster.

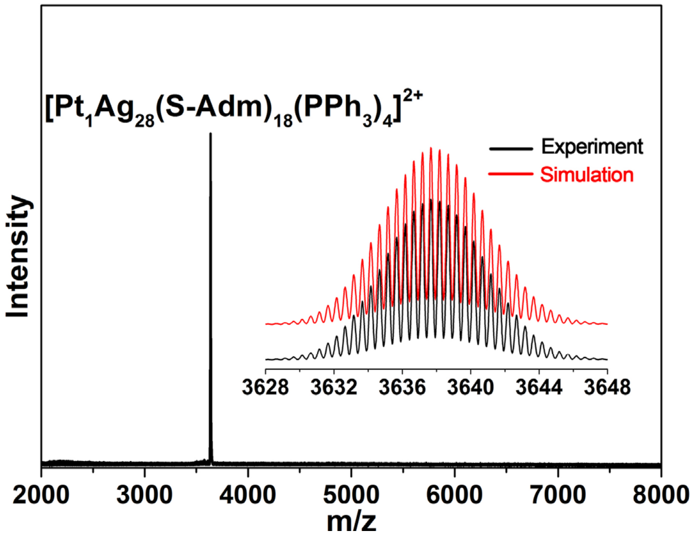

Fig. S4. ESI-MS mass spectrum of Pt_1Ag_{28} in positive-mode. Insets: the experimental (black) and simulated (red) isotope patterns of Pt_1Ag_{28}.

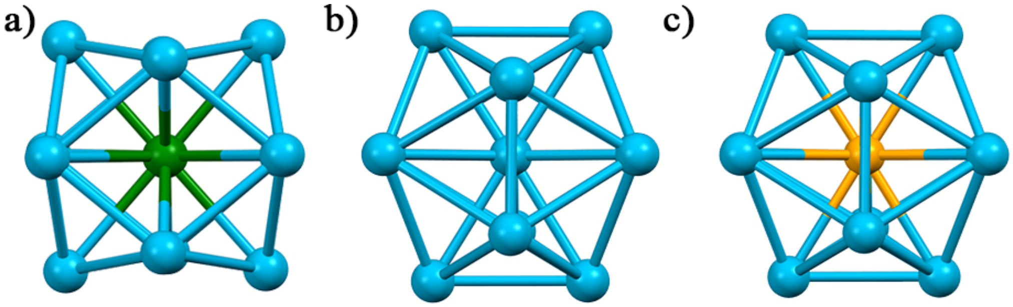

Fig. S5. The M_{13} kernel of a) Pt_1Ag_{28}, b) Ag_{29} and c) Au_1Ag_{28} nanoclusters. Color legend: green sphere, Pt; cerulean sphere, Ag.

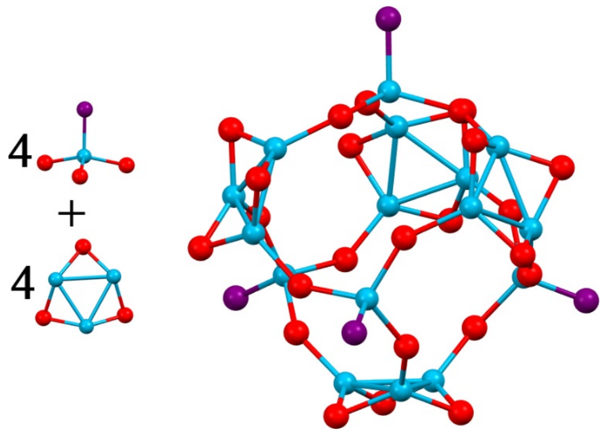

Fig. S6. The motifs in Ag_{29} and Au_1Ag_{28} nanoclusters. The overall motif could be separated into 4 $Ag_1S_3P_1$ and 4 Ag_3S_3 motifs. Color legend: cerulean sphere, Ag; red sphere, S; purple sphere, P.

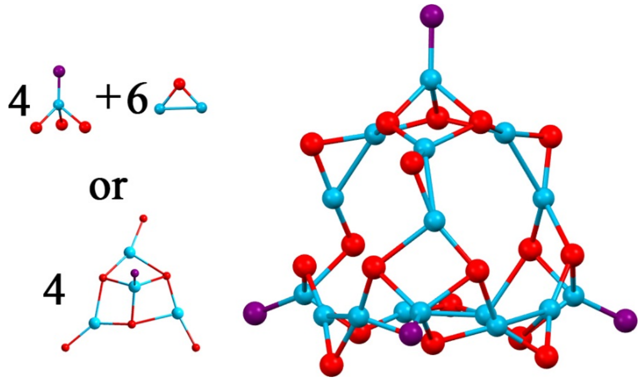

Fig. S7. The motifs in Pt_1Ag_{28} nanocluster. The overall motif could be separated into 4 $Ag_1S_3P_1$ and 6 Ag_2S_1 motifs or 4 $Ag_4S_6P_1$ motifs sharing 6 S atoms. Color legend: cerulean sphere, Ag; red sphere, S; purple sphere, P.

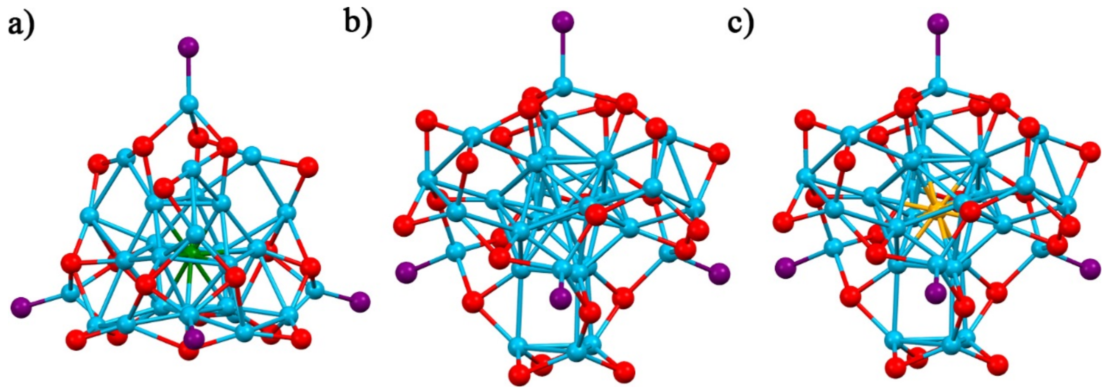

Fig. S8. Total structure of a) Pt_1Ag_{28}, b) Ag_{29} and c) Au_1Ag_{28} nanoclusters. Color legend: green sphere, Pt; cerulean sphere, Ag; orange sohere, Au; red sphere, S; purple sphere, P. For clarity, C and H are not shown.

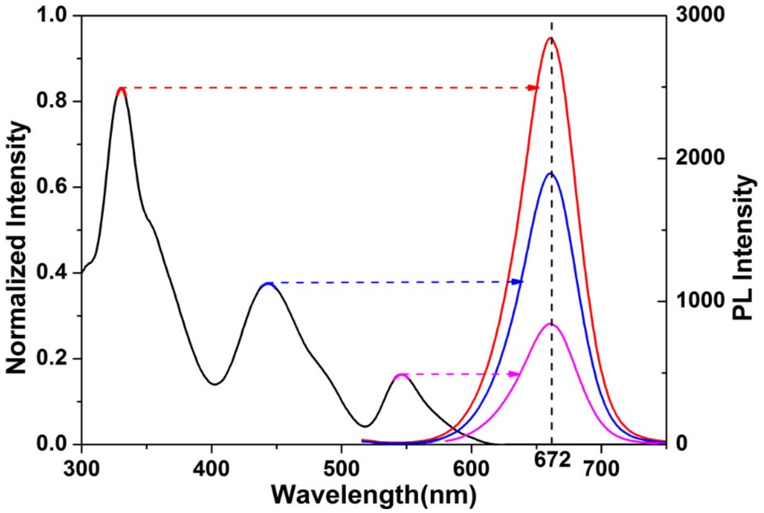

Fig. S9. Photoluminescence properties of $Pt_1Ag_{28}(S\text{-}Adm)_{18}(PPh_3)_4$. Excitation spectrum (left) and emission spectra (right) at different excitation wavelengths, as indicated by the arrows.

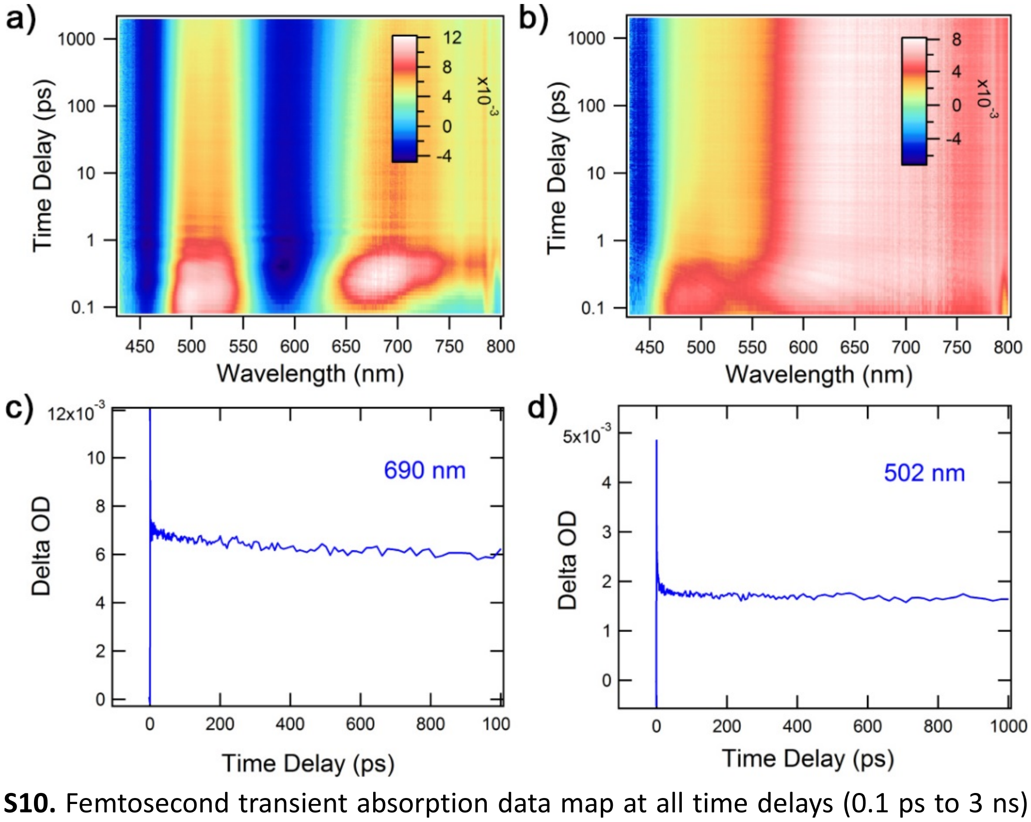

Fig. S10. Femtosecond transient absorption data map at all time delays (0.1 ps to 3 ns) and probe wavelengths for a) Pt_1Ag_{24} and b) Pt_1Ag_{28} nanoclusters with excitation of 360 nm. c, d) Kinetic traces at selected wavelengths between -1 ps and 1 ns for c) Pt_1Ag_{24} and d) Pt_1Ag_{28}.

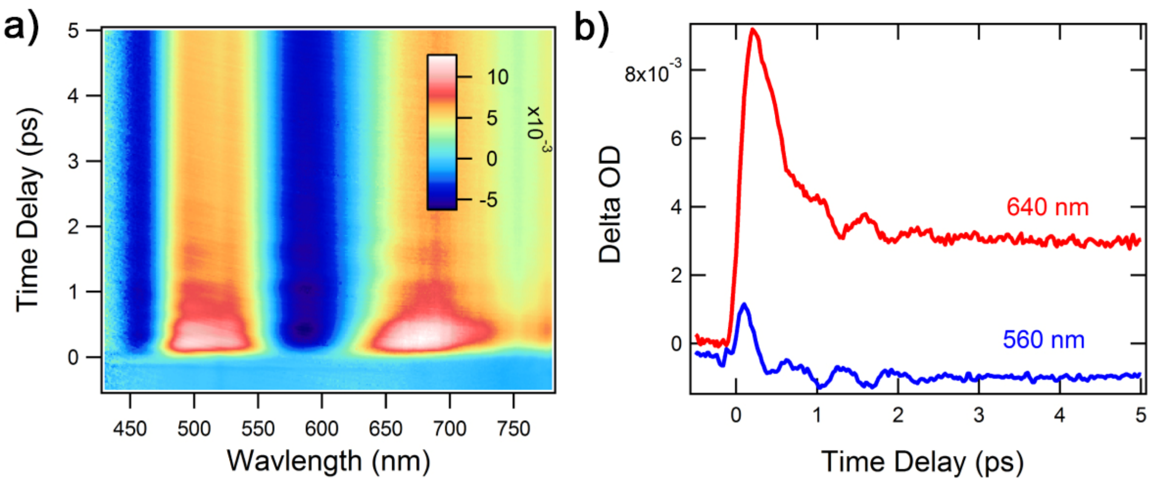

Fig. S11. a) Femtosecond transient absorption data map of Pt_1Ag_{24} at early time delays (-0.5 ps to 5 ps). b) Coherent oscillations observed in kinetic traces at selected wavelengths.

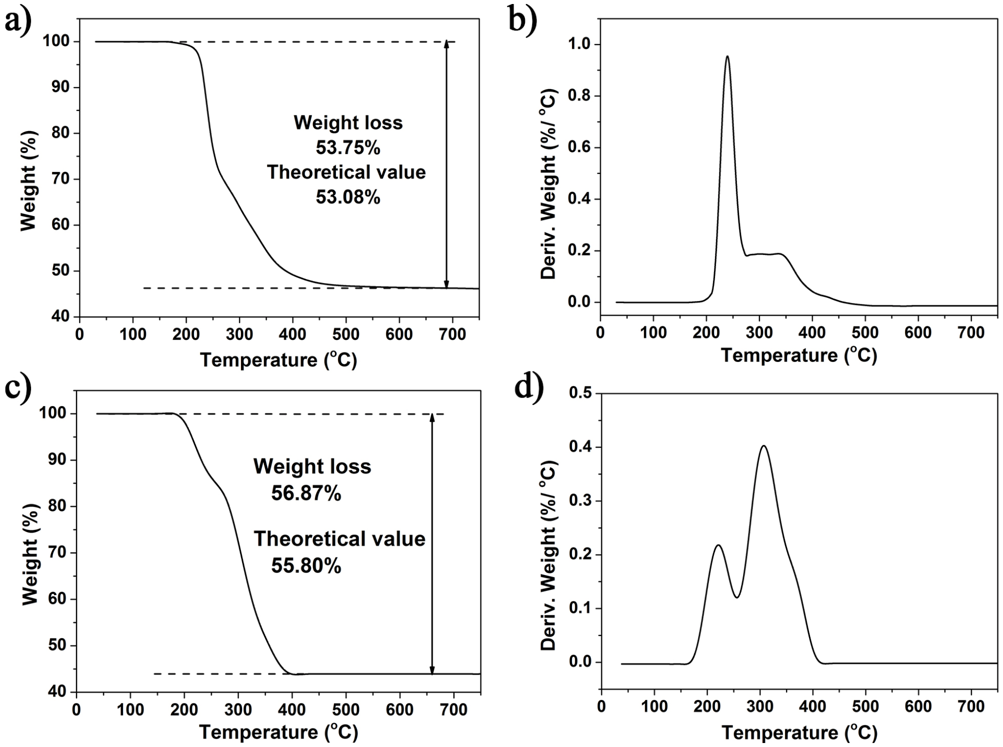

Fig. S12. a) TGA curve and b) the derivative of Pt_1Ag_{24}; c) TGA curve and d) the derivative of Pt_1Ag_{28}.

Table S1. The atom ratio of Pt and Ag in Pt_1Ag_{28}(S-Adm)$_{18}$(PPh_3)$_4$ nanocluster calculated from inductively coupled plasma (ICP) and X-ray photoelectric spectroscopy (XPS) measurements.

	Pt atom	Ag atom
ICP Experiment ratio of Pt_1Ag_{28}(S-Adm)$_{18}$(PPh_3)$_4$	3.9%	96.1%
XPS Experiment ratio of Pt_1Ag_{28}(S-Adm)$_{18}$(PPh_3)$_4$	3.5%	96.5%
Theoretical ratio of Pt_1Ag_{28}(S-Adm)$_{18}$(PPh_3)$_4$	1/29(3.45%)	28/29(96.55%)

Table S2. Structural analysis of Ag_{29}, Au_1Ag_{28} and Pt_1Ag_{28} nanoclusters.

	M(center)-Ag lengths in the kernel (Å)	Ag-S lengths on the kernel-shell (Å)	Ag-Ag lengths on the kernel-shell (Å)	P-Ag-S angles on the same motifs (^{o})
Ag_{29}	2.755-2.772	2.433-2.474	3.077-3.158	104.12-109.19
Averages	2.765	2.459	3.111	106.91
Au_1Ag_{28}	2.745-2.775	2.425-2.472	3.123-3.161	104.50-108.63
Averages	2.761	2.444	3.141	105.94
Pt_1Ag_{28}	2.768-2.797	2.438-2.498	2.934-3.157	119.28-131.46
Averages	2.783	2.472	3.104	126.80

Table S3. Excited state lifetimes of silver and doped silver nanoclusters.

Sample	Excited State Lifetimes
Ag_{25}[1]	1.1 µs
Pt_1Ag_{24}	1.9 µs
Ag_{29}[2]	350 ns
Pt_1Ag_{28}	300 ns, 2.9 µs

Table S4: Crystal data and structure refinement for the $Pt_1Ag_{28}(S\text{-}Adm)_{18}(PPh_3)_4$ nanocluster.

Compound reference	$Pt_1Ag_{28}(S\text{-}Adm)_{18}(PPh_3)_4$	
Empirical formula	C252H330Ag28PtP4S18	
Formula weight	7275.55	
Temperature	143 K	
Wavelength	1.54178 Å	
Crystal system	Monoclinic	
Space group	C 2/c	
Unit cell dimensions	A=50.8135 (18) Å	α=90°
	B=29.3497(8)Å	β=110.274(4)°
	C=49.1614(16)Å	γ=90°
Volume	68775(4) Å^3	
Z	8	
Density (calculated)	1.405 Mg m^{-3}	
Absorption coefficient	14.666 mm^{-1}	
F(000)	28272	
Crystal size	0.05 × 0.03 × 0.02 mm^3	
Theta range for data collection	3.01 to 55.95°	
Index ranges	-63<=h<=59, -34<=k<=36, -57<=l<=61	
Reflections collected	239535	
Independent reflections	67855	
Absorption correction	Multi scan	
Refinement method	SHELXL-2014/7 (Sheldrick 2014/7)	
Data / restraints / parameters	67855 / 8 / 2728	
Goodness-of-fit on F2	1.048	
Final R indices [I>2sigma(I)]	R1=0.0333, wR2=0.0845	
R indices (all data)	R1=0.0432, wR2=0.0867	

References.

[1] Bootharaju, M. S., Joshi, C. P., Parida, M. R., Mohammed, O. F. & Bakr, O. M. Templated atom-precise galvanic synthesis and structure elucidation of a $[Ag_{24}Au(SR)_{18}]$ nanocluster. *Angew. Chem. Int. Ed.* **55**, 922-926 (2016).

[2] Soldan, G., Aljuhani, M. A., Bootharaju, M. S., AbdulHalim, L. G., Parida, M. R., Emwas, A. –H., Mohammed, O. F. & Bakr, O. M. Gold doping of silver nanoclusters: a 26-fold enhancement in the luminescence quantum yield. Angew. Chem. Int. Ed. 55, 5749-5753 (2016).

[3] AbdulHalim, L. G., Bootharaju, M. S., Tang, Q., Gobbo, S. D., AbdulHalim, R. G., Eddaoudi, M., Jiang, D. –en. & Bakr, O. M. $Ag_{29}(BDT)_{12}(TPP)_4$: a tetravalent nanocluster. J. Am. Chem. Soc. 137, 11970-11975 (2015).
